# Far and Few Between: Early Onset Multiple Myeloma in a 26-Year-Old Female

**DOI:** 10.7759/cureus.9588

**Published:** 2020-08-06

**Authors:** Abdul Wali Khan, Asad Ali Khan, Talal Almas, Muhammad Ishaq, Irfan Ullah

**Affiliations:** 1 Internal Medicine, Hayatabad Medical Complex, Peshawar, PAK; 2 Internal Medicine, Khyber Teaching Hospital, Peshawar, PAK; 3 Internal Medicine, Royal College of Surgeons in Ireland, Dublin, IRL; 4 Internal Medicine, Kabir Medical College, Peshawar, PAK; 5 Internal Medicine, Naseer Teaching Hospital, Peshawar, PAK

**Keywords:** multiple myeloma, early onset

## Abstract

Multiple myeloma (MM), a common hematologic malignancy, is predominantly a disease of old age with a median age at diagnosis hovering around 70 years. Medical literature remains largely bereft of reports of the ailment in young female patients. We chronicle the case of a 26-year-old female patient who presented with a history of fever, weight loss, constipation, frequent infections, and back and chest pain. Laboratory workup divulged underlying anemia, renal impairment, increased calcium, and significant urinary proteins, insinuating a diagnosis of MM. Subsequent bone marrow examination confirmed a diagnosis of MM. Following treatment with a concoction of dexamethasone, cyclophosphamide and bortezomib, the patient improved rapidly, and her clinical symptoms abated. This article further accentuates the need for physicians to be cognizant of the possibility of early-onset MM in unusually young patients.

## Introduction

Multiple myeloma (MM) arises from an aberrant proliferation of neoplastic monoclonal plasma cells in the bone marrow and produces nonfunctional intact immunoglobulins and/or immunoglobulin light chains. An exceedingly common hematologic malignancy, it accounts for roughly 1% of all cancers and 10% of all hematologic neoplasms [1]. The median age at diagnosis hovers around 70 years, with the risk increasing proportionately with advancing age [1]. Interestingly, MM is seldom observed in younger patients; only a few cases of MM afflicting younger patients have been reported thus far.

Common signs and symptoms elicited by the disease include hypercalcemia, renal failure, anemia, bone pain secondary to osteolytic bone lesions, and weight loss [2]. Although much less common, MM can also present with more sinister signs and symptoms such as hepatosplenomegaly and restrictive cardiomyopathy [3]. The hypercalcemia elicited by MM often contributes towards the onset of renal disease that remains ubiquitous in patients afflicted with the ailment [4]. Furthermore, light chain cast nephropathy and light chain amyloidosis, as observed in MM, can further herald the onset of chronic renal disease in affected patients.

A patient suspected to have MM is evaluated through extensive laboratory workup, including peripheral smear, serum calcium, albumin, serum protein electrophoresis, immunofixation, serum-free light chain analysis, and urine protein electrophoresis. Additionally, skeletal radiographs are evaluated for the presence of osteolytic bone lesions that are typical of MM. Nevertheless, bone marrow biopsy remains the diagnostic investigative modality for MM. We hereby delineate a rare case of MM diagnosed in a 26-year-old female. Imperatively, the disease progressed to renal impairment in our patient. We also elucidate herein the overarching need for a timely diagnosis and prompt intervention in order to thwart the onset of renal impairment.

## Case presentation

**Figure 1:**
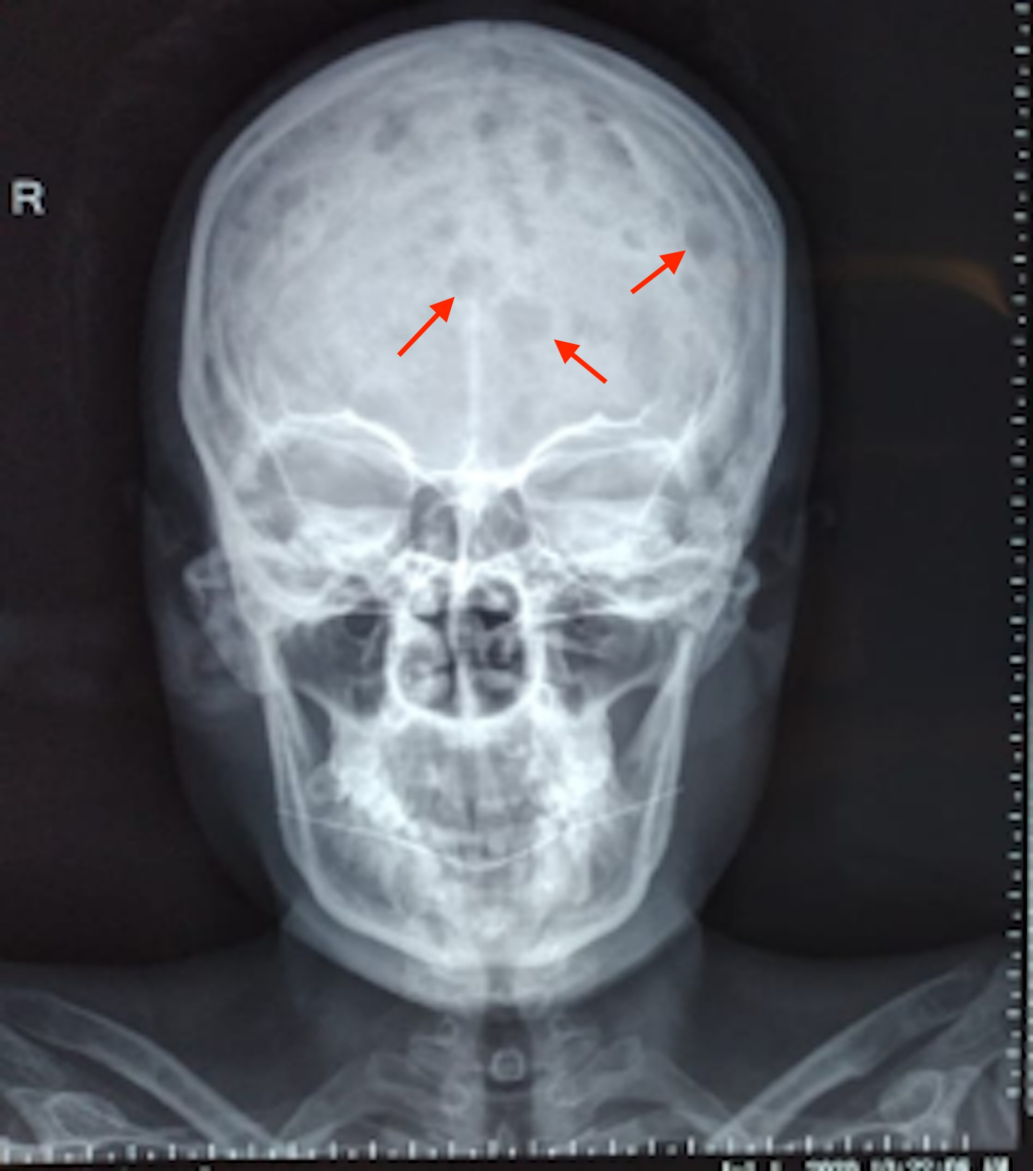
A skull radiograph depicting multiple punched out lytic lesions (arrows) indicative of multiple myeloma

**Figure 2:**
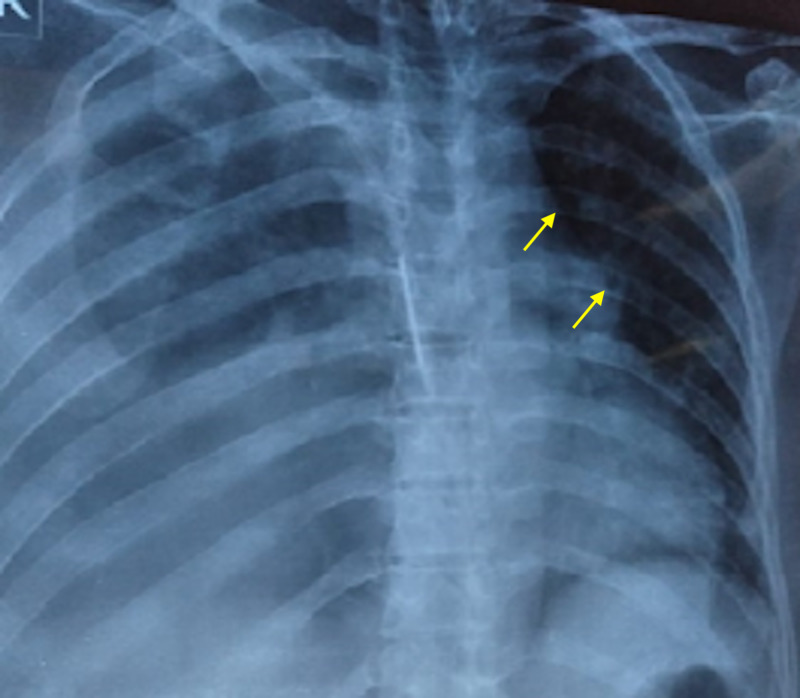
A chest radiograph elucidating multiple small lytic lesions of the ribs, which are suggestive of multiple myeloma

**Figure 3:**
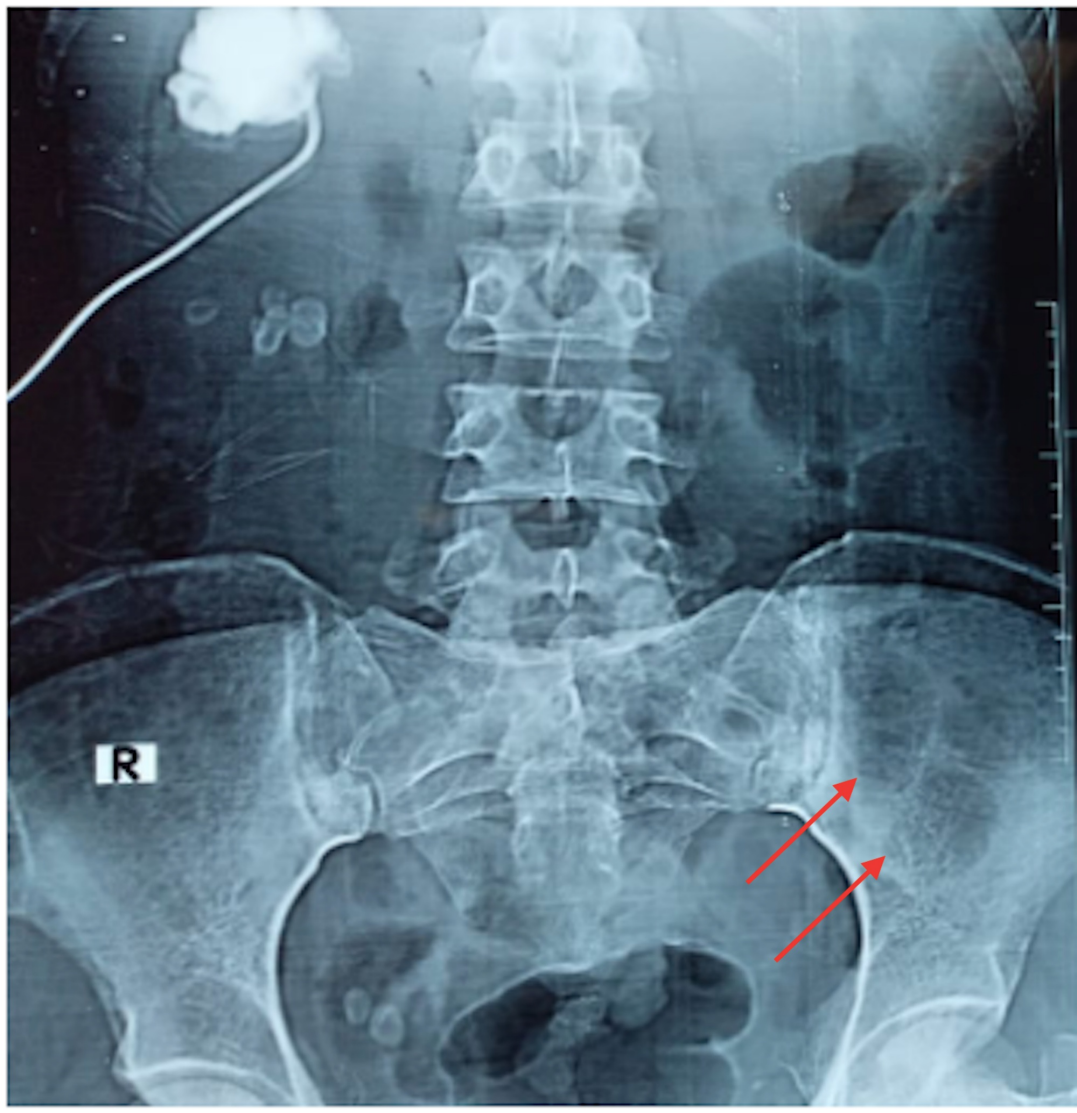
A radiograph of the pelvis showing lytic lesions afflicting the left ileum bone, suggestive of multiple myeloma

We delineate the case of a 26-year-old female who presented to us with fever, constipation, back pain and chest pain for the past one month. On further evaluation, the patient disclosed that she had been having intermittent headaches, pelvic pain, occasional gum bleeding, asthenia, loss of appetite, and weight loss. Additionally, the patient also reported a history of recent onset frequent upper respiratory tract infections and urinary tract infections. Of note, the patient had consulted her primary health care facility in the preceding month with complaints of flank pain, headache, and fatigue, for which she had been offered over-the-counter analgesics. The patient had subsequently undergone basic laboratory investigations. These investigations were significant for anemia (hemoglobin= 9 g/dL) and deranged renal function tests (creatinine = 12 mg/dL and urea = 201 mg/dL). In the days that followed, her condition considerably deteriorated, with the development of right obstructive uropathy and worsening renal function. She was thus admitted to the nephrology unit of our hospital and underwent hemodialysis; a double-J (DJ) stent was passed to relieve her urinary outflow obstruction. Further workup at our hospital revealed a hemoglobin of 8.1 g/dL (normal range = 12-15.5 g/dL) , white blood cell count of 19,750 cells/μL (normal range = 4,500-11,000 cells/μL) , serum calcium (corrected) of 11.20 mg/dL (normal range = 8.6-10.3 mg/dL), serum creatinine of 8 mg/dL (normal range = 0.84-1.21 mg/dL) , blood urea of 200 mg/dL (normal range = 7-20 mg/dL), urinary proteins of 380.1 mg/dL (normal range = 0-14 mg/dL), and alkaline phosphatase level of 256 IU/L (normal range = 20-140 IU/L) . Furthermore, her coagulation profile was also deranged with a prothrombin time (PT) of 28.1 seconds (normal range = 11-13.5 seconds), international normalized ratio (INR) of 2.4 (normal range = less than 1.1), and an activated partial thromboplastin time (aPTT) of 60.9 seconds (normal range = 30-40 seconds). To confirm the suspicion of MM, a radiographic skeletal survey was carried out, which divulged multiple lytic, punched out lesions in the skull, ribs, and pelvic bones (Figures 1, 2, 3).

Thereafter, a bone marrow examination was ordered and divulged a hypercellular bone marrow with focal areas infiltrated by 80% plasma cells, confirming the diagnosis of MM. 

The patient was then commenced on hemodialysis, which resulted in a significant drop in serum creatinine and serum urea levels. Furthermore, the patient was initiated on broad spectrum antibiotics; her fever subsided within two days of therapy commencement. Treatment for MM using a concoction of dexamethasone, cyclophosphamide, and bortezomib was thus initiated, and a follow-up with the consultant oncologist was planned.

## Discussion

MM is predominantly a disease of the elderly, with the median age at diagnosis hovering at 70 years [5]. A meticulous perusal of oncological literature shows that merely 0.02% of the total cases of MM are diagnosed in patients below the age of 30, alluding to the remarkable rarity of our case [6]. To date, only a few cases of MM in patients younger than 30 years of age have been reported [7,8]. Interestingly, certain studies have noted that MM in younger individuals is usually more aggressive and also more responsive to treatment [9,10].

Monoclonal gammopathy of undetermined significance (MGUS) and smoldering MM are two clinically similar differential diagnoses of MM. Due to their overlapping presentations, these pathologies can often obscure the timely diagnosis of MM. There are three criteria that must be met in order to yield a confirmed diagnosis of MM. Firstly, there should be greater than 10% monoclonal plasma cells on bone marrow examination. Secondly, there should be sufficient evidence of end-organ damage, which usually manifests in the form of hypercalcemia, anemia, renal failure, bone disease, amyloidosis, and recurrent infections. Thirdly, a urine or plasma protein electrophoresis should reveal an M-spike. Additionally, a deranged free light chain ratio is also observed in patients afflicted with MM [11]. In our case, bone marrow examination revealed an 80% plasma cell infiltration. Additionally, there was prominent renal impairment, a history of frequent respiratory infections, and radiological findings highly suggestive of MM. Moreover, in our case, serum kappa light chain levels were also increased. These findings are in concordance with the established diagnostic criteria. Taken together, these findings insinuate an undeniable diagnosis of MM.

A study conducted at Mayo Clinic showed that around 73% of patients with MM present with some degree of anemia [2]. In such cases, anemia of chronic disease is the most common cause. To treat the underlying anemia, blood transfusions and erythropoietin analogs are employed and remain the mainstay of therapy. The subject in our study had a hemoglobin level of 8.1 g/dL, which improved to 12 g/dL after transfusion, thereby demonstrating the efficacy of transfusions in addressing anemia secondary to MM. Additionally, hemostatic abnormalities, including bleeding tendencies and thrombotic risks, are frequently encountered in these patients. There is thus a need to yield a prompt diagnosis of MM so that these underlying abnormalities can be attended to and in turn management. Contrarily, a dilatory detection of the true underlying etiology can result in the advancement of renal damage, thereby portending adverse disease outcomes. Furthermore, an unusually young age at presentation should not exclusively preclude the physicians’ suspicion of MM.

## Conclusions

Although it predominantly affects the elderly population, multiple myeloma can also rarely present in much younger patients. A meticulous evaluation through the means of blood workup, radiological investigations, and bone marrow examination remains pivotal in ascertaining an underlying diagnosis of multiple myeloma. When it presents in younger patients, multiple myeloma is noted to have a more aggressive disease course; however, a good response to therapy as observed in younger patients can effectively thwart disease progression. A timely diagnosis therefore remains imperative in yielding favorable disease outcomes in this population.
